# CYP2J2 Is a Diagnostic and Prognostic Biomarker Associated with Immune Infiltration in Kidney Renal Clear Cell Carcinoma

**DOI:** 10.1155/2021/3771866

**Published:** 2021-06-23

**Authors:** Xiong Zou, Zengnan Mo

**Affiliations:** ^1^Department of Urology, The First Affiliated Hospital of Guangxi Medical University, 530021 Nanning, Guangxi, China; ^2^Center for Genomic and Personalized Medicine, Guangxi Medical University, 530021 Nanning, Guangxi, China; ^3^Guangxi Collaborative Innovation Center for Genomic and Personalized Medicine, 530021 Nanning, Guangxi, China; ^4^Guangxi Key Laboratory for Genomic and Personalized Medicine, Guangxi Key Laboratory of Colleges and Universities, 530021 Nanning, Guangxi, China

## Abstract

Cytochrome P450 family 2 subfamily J member 2 (CYP2J2), a member of the monooxygenase cytochrome P450 (CYP) family and the only member of the human CYP2J subfamily, has many functions, including regulation of oxidative stress, inflammation, apoptosis, and immune responses. However, its role in cancer development has not been clearly elucidated. In this study, expression levels of CYP2J2 in various cancer types were determined using the Oncomine, the Gene Expression Profiling Interactive Analysis (GEIPA), DriverDBv3, UALCAN, and Tumor Immune Estimation Resource (TIMER) databases. The prognostic value of CYP2J2 for KIRC was analyzed using GEPIA, UALCAN, OSkirc, and DriverDBv3 databases. We evaluated the expression levels of CYP2J2 transcript, protein, and promoter methylation at different clinical characteristics in KIRC through the UALCAN database. Simultaneously, CYP2J2 network-related functions were evaluated using the GeneMANIA interactive tool while the biological processes involved in CYP2J2 and its interactive genes were investigated through Metascape and FunRich. Then, we used TIMER to determine the correlation between CYP2J2 expression levels and immune infiltration levels in KIRC. In KIRC, the CYP2J2 gene, RNA, and protein were found to be overexpressed. However, the methylation level of CYP2J2 promoter in KIRC was lower than in normal tissues. Surprisingly, elevated expression levels of CYP2J2 exhibited better prognostic outcomes in KIRC. Evaluation of protein-protein interaction networks and biological processes revealed that CYP2J2 was principally involved in immune responses, apoptosis, and other metabolic processes. Moreover, we found that the expression levels of CYP2J2 were positively correlated with infiltration levels of B cells, CD8 + T cells, neutrophils, and dendritic cells in KIRC. Therefore, we speculated that the overexpression of CYP2J2 prolonged the survival outcome of KIRC patients, which may be related to the change of tumor immune microenvironment. Moreover, all these new understandings of CYP2J2 may provide important value for the early diagnosis and new targeted drug therapy of KIRC.

## 1. Introduction

Renal cell carcinoma (RCC), a tumor whose origin is the renal epithelium [1], can be grouped into various subtypes based on its histological characteristics, with KIRC accounting for the vast majority of renal cell carcinoma subtypes. KIRC, a metabolic disease histologically characterized by lipid accumulation and storage [2], has been reported to be the most frequent cause of kidney cancer-associated mortalities [3]. Globally, KIRC is associated with 175,000 annual mortalities, with about 30-35% of patients undergoing surgery presenting with distant metastases [4]. Patients with relapsed or distant metastases of KIRC exhibit poor prognostic outcomes, with shorter median survival times of 21 months and 13 months [5], respectively. Several studies have suggested tumor lymph node metastasis (TNM) staging system is the main prognostic factor for KIRC [5–7]. However, prognostic stratification by molecular markers (such as expression of specific genes) can improve the accuracy of prognostic prediction [8]. Therefore, identifying early diagnostic markers and prognostic molecular biomarkers will enhance early KIRC diagnosis, thereby informing early and active treatment.

Through the Oncomine, GEIPA, DriverDBv3, and TIMER databases, we found that CYP2J2 was differentially expressed in multiple tumors, with its expression in KIRC being most significantly elevated. The CYP2J2 gene encodes an enzyme responsible for the oxidative metabolism of various exogenous and endogenous compounds and is involved in various physiological and pathological processes in the human body [9]. It has been reported that CYP2J2 plays various roles in the human body, including anti-inflammatory [10], regulation of cardiovascular functions [11], improving metabolism [11], and in immune regulation [12]. In addition, CYP2J2 has been associated with the occurrence and development of many tumor types [13]; however, its role in KIRC pathogenesis has not been reported.

This study is aimed at evaluating the expression levels of CYP2J2 in KIRC as well as determining its value in predicting survival outcomes for KIRC patients through multiple databases, including Oncomine, GEPIA, UALCAN, OSkirc, DriverDBv3, and TIMER. In order to further speculate the possible mechanism of CYP2J2 in KIRC, we used GeneMANIA, Metascape, and FunRich to investigate the functional networks involving CYP2J2 as well as the biological processes involving CYP2J2 interactive genes. At the same time, we evaluated the relationships between CYP2J2 expression and immune cell infiltration levels in KIRC through the TIMER database. This study elucidates on the correlation between CYP2J2 expression levels and KIRC prognosis and provides a potential early diagnostic biomarker and therapeutic target for KIRC.

## 2. Materials and Methods

### 2.1. Oncomine

Oncomine (https://www.oncomine.org/resource/main.html), a database dedicated to collecting and analyzing cancer-related data [14], has 715 datasets from 86733 samples [15]. This database was used to determine the expression levels of CYP2J2 in a variety of cancers using the following criteria: *p* value of 1*E* − 4, fold change as 2, gene rank as top 10%, and data type as all (DNA and mRNA).

### 2.2. GEPIA

GEPIA (http://gepia.cancer-pku.cn/) generates gene expression profiles of multiple cancer types and pairs of normal samples using the TCGA and GTEx databases [16]. GEPIA was used to determine the expression levels of the CYP2J2 gene in a variety of cancers. Moreover, we evaluated the correlation between CYP2J2 expression levels and overall survival (OS) as well as disease-free survival (DFS) for KIRC patients. Regarding the survival curve, *p* < 0.05 was considered statistically significant.

### 2.3. UALCAN

UALCAN (http://ualcan.path.uab.edu/analysis.html) is a website with multiple functions for analyzing and mining TCGA databases, thereby allowing users to verify the expression levels of genes in various cancer types, draw graphs describing gene expression and patient survival information, and to assess epigenetic regulation of gene expression by promoter methylation [17]. We determined the expression levels of the CYP2J2 gene, transcript, protein, and promoter methylation in KIRC and the effect of CYP2J2 on the patients' survival time through UALCAN. Regarding the survival curve, *p* < 0.05 was considered statistically significant.

### 2.4. OSkirc

OSkirc (http://bioinfo.henu.edu.cn/KIRCCombined) is a free and fast online tool that enables users to easily investigate the prognostic value of genes associated with KIRC [18]. Through this database, we used the following criteria to detect the effect of CYP2J2 on the overall survival of KIRC patients: the data source from a combination of GSE22541, GSE29609, GSE3, and TCGA; split patients by upper 50% VS lower 50% and upper 30% VS lower 30%. Regarding the survival curve, *p* < 0.05 was considered statistically significant.

### 2.5. DriverDBv3

DriverDBv3 (http://driverdb.tms.cmu.edu.tw/) is a powerful cancer multiomics database for copy number variations, miRNA expression levels, RNA expression, methylation, and somatic mutations among other clinical data [19]. One of the many functions of the DriverDBv3 database is that it allows users to analyze the association between cancer and genes through “Cancer,” “Gene,” and “Customized analysis.” In this study, DriverDBv3 was used to assess the expression levels of CYP2J2 in different cancer types and to evaluate its prognostic value in KIRC patients. Regarding the survival curve, *p* < 0.05 was considered statistically significant.

### 2.6. GeneMANIA

GeneMANIA (http://genemania.org/) is an easy-to-use website for establishing protein-protein interactions (PPI), protein-DNA interactions, and genetic interactions [20]. In this study, the functions and networks of the CYP2J2 protein were determined through GeneMANIA.

### 2.7. Metascape and FunRich

Metascape (http://metascape.org/gp/index.html) is an effective tool for comprehensive genomics analysis in the era of big data. It integrates functional enrichment, gene annotation, and interactive group analysis [21]. FunRich (3.1.3 exe) is a user-friendly bioinformatics tool for performing various analyses on generated datasets [22]. We obtained the interactive genes of CYP2J2 through the GeneMANIA network. Then, these genes were inputted into Metascape and FunRich for functional evaluations.

### 2.8. TIMER

TIMER (https://cistrome.shinyapps.io/timer/) is a simple and practical cancer web server that allows users to evaluate the immunological, genomic, and clinical features of tumors by inputting function-specific parameters [23]. In this study, TIMER was used to determine the expression levels of CYP2J2 in various cancers and to evaluate the correlation between the expression levels of CYP2J2 and immune infiltration levels in KIRC.

## 3. Results

### 3.1. Expression Levels of CYP2J2 in Different Cancer Types

**Figure 1:**
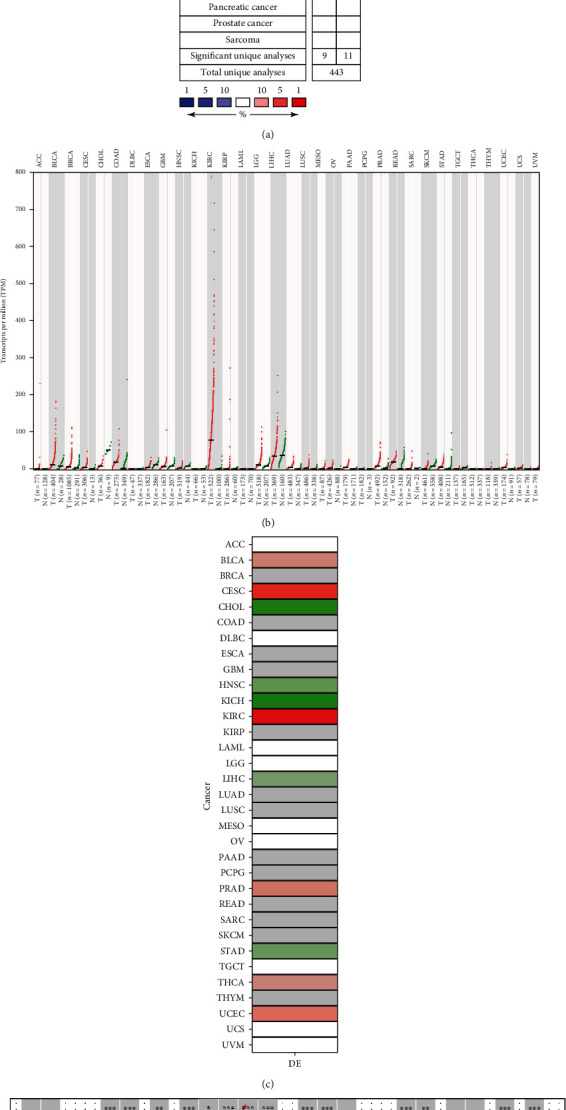
Expression levels of CYP2J2 in various human cancers. (a) Expression profiles of the CYP2J2 gene in tumor and paired normal tissue samples from the Oncomine database. (b) Expression profiles of the CYP2J2 transcript in different cancer types and paired of normal tissues from the GEPIA database. (c and d) Expression levels of the CYP2J2 gene in different cancer types compared to corresponding normal tissues from the DriverDBV3 and TIMER database. CYP2J2: Cytochrome P450 family 2 subfamily J member 2.

Using the Oncomine database, which contained total unique analyses of 443 about CYP2J2, we detected diversities in CYP2J2 gene expression profiles between tumor and matched normal tissues. Compared to normal tissues, elevated expression levels of CYP2J2 were observed in bladder, kidney, lung, lymphoma, and ovarian cancer types, while suppressed expression levels of CYP2J2 were observed in cervical, colorectal, esophageal, head and neck, and liver cancer types (Figure 1(a)). Although the expression of CYP2J2 was elevated in a variety of cancers, it was found that elevated CYP2J2 expression levels were most significant in kidney cancer. Then, we analyzed the RNA sequencing data of CYP2J2 through GEPIA. Compared to matched normal tissues, expression levels of CYP2J2 transcripts per million in KIRC were most significant among various tumors (Figure 1(b)). Moreover, expression levels of the *CYP2J2* gene in the DriverDBv3 and TIMER database were also the highest in KIRC (Figures 1(c) and 1(d)). These findings showed that the CYP2J2 expression level in KIRC was significantly upregulated compared with normal tissues. Therefore, CYP2J2 may have a potential diagnostic value for KIRC and the correlation between CYP2J2 and KIRC was worth further exploring.

### 3.2. Potential Prognostic Values of CYP2J2 in KIRC

**Figure 2:**
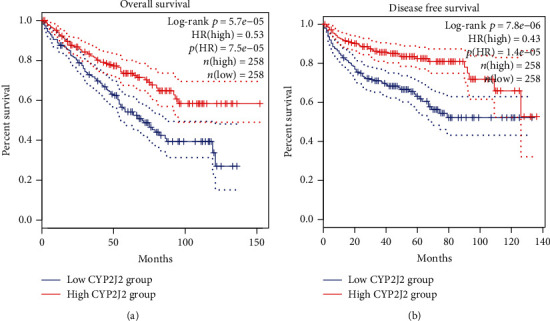
Comparisons of the effects of high and low expression levels of CYP2J2 on survival time of KIRC patients using GEPIA database. (a and b) Elevated expression levels of CYP2J2 were associated with longer OS and DFS outcomes for KIRC patients. OS: overall survival; DFS: disease free survival; KIRC: kidney renal clear cell carcinoma.

To determine whether CYP2J2 expression levels are correlated to the prognosis of cancer patients, we evaluated the prognostic value of CYP2J2 in cancer using GEPIA. We found that, even though CYP2J2 expression levels were upregulated or downregulated in various tumor types, CYP2J2 expression levels only had significant correlations with the overall survival time (OS) and disease-free survival (DFS) of KIRC. Moreover, the results indicated that overexpression of CYP2J2 in KIRC prolonged the OS and DFS (Figure 2). Based on the unique prognostic value of CYP2J2 in KIRC, we further analyzed the relationship between CYP2J2 and KIRC using other databases.

**Figure 3:**
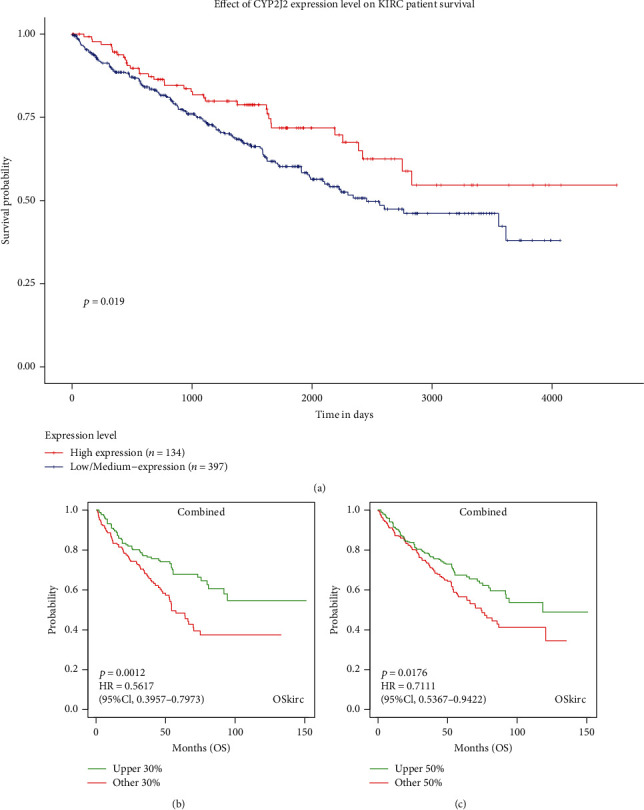
Effect of CYP2J2 expression levels in UALCAN (a) and OSkirc (b and c) database on the survival of KIRC patients. Overexpression of CYP2J2 prolonged the survival time of KIRC patients.

**Figure 4:**
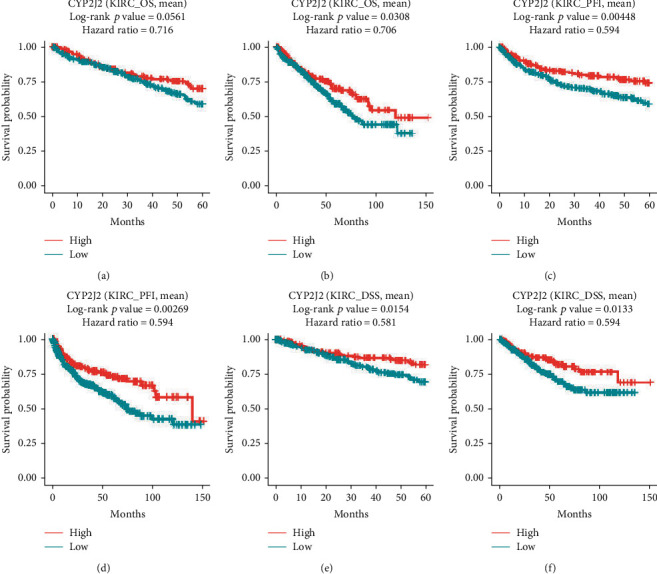
Prognostic value of CYP2J2 expression in KIRC patients was evaluated based on DriverDBv3 database. (a and b) Overexpression of CYP2J2 prolonged OS of KIRC patients. (c–f) Overexpression of CYP2J2 prolonged PFI and DSS of KIRC patients. OS: overall survival; PFI: platinum-free treatment interval; DSS: disease specific survival.

From the UALCAN database, it was found that CYP2J2 overexpression was associated with longer survival outcomes in KIRC patients (Figure 3(a)). Furthermore, we combined the GSE22541, GSE29609, GSE3, and TCGA datasets from OSkirc to verify the effect of CYP2J2 on the survival outcome of KIRC patients, and the results also showed that overexpression of CYP2J2 prolonged OS in patients with KIRC (Figures 3(b) and 3(c)). For the DriverDBv3 database, overexpression of CYP2J2 in KIRC predicted better prognostic outcomes when compared to its low expression level (Figure 4), which was basically consistent with the results obtained from GEPIA, UALCAN, and OSkirc databases. Therefore, it can be concluded that the expression levels of CYP2J2 had a very momentous potential prognostic value for KIRC.

### 3.3. Reanalysis of CYP2J2 Expression in KIRC

**Figure 5:**
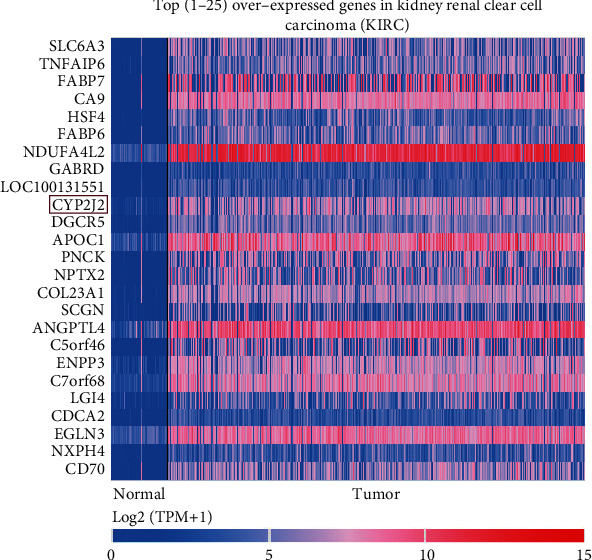
CYP2J2 gene is one of the top [1–25] overexpressed genes in KIRC tumor tissues compared to corresponding normal tissues.

**Figure 6:**
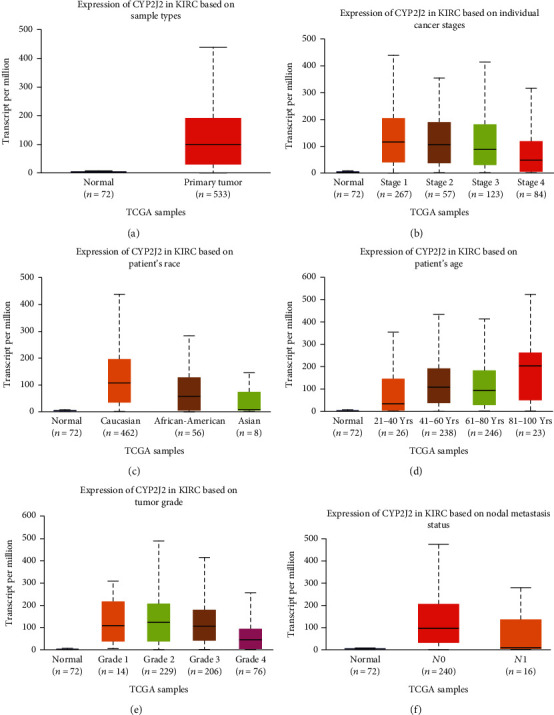
Expression of CYP2J2 mRNA in KIRC was evaluated by box plot using the UALCAN database. (a–f) Box plots showing the association of sample types, cancer stage, patient's race, age, tumor grade, and nodal metastasis status with CYP2J2 expression in KIRC.

To better elucidate on the relationship between CYP2J2 and KIRC, we determined the expression levels of the CYP2J2 gene in KIRC using the UALCAN database. The CYP2J2 gene was one of the top [1–25] overexpressed gene in KIRC (Figure 5), which further indicated that expression levels of the CYP2J2 gene in KIRC tumor tissues were significantly higher than those of normal tissues. Further analyses revealed that the expression levels of the CYP2J2 transcript in KIRC tumor tissues were significantly higher compared to matched normal samples (Figure 6(a)). Then, the UALCAN database was used to assess various clinical characteristics of KIRC, including cancer stage, patients' race, age, tumor grade, and nodal metastasis status. Further analyses showed that expression levels of the CYP2J2 transcript in KIRC patients were higher in characteristics such as cancer stage 1, Caucasian race, age from 81 to 100 years old, grade 2, and *N*0 than other clinical characteristics (Figures 6(b)–6(f)). These findings imply that overexpression of CYP2J2 can be used as an early diagnostic marker of KIRC.

### 3.4. Potential Diagnostic Values of CYP2J2 Protein Expression in KIRC

**Figure 7:**
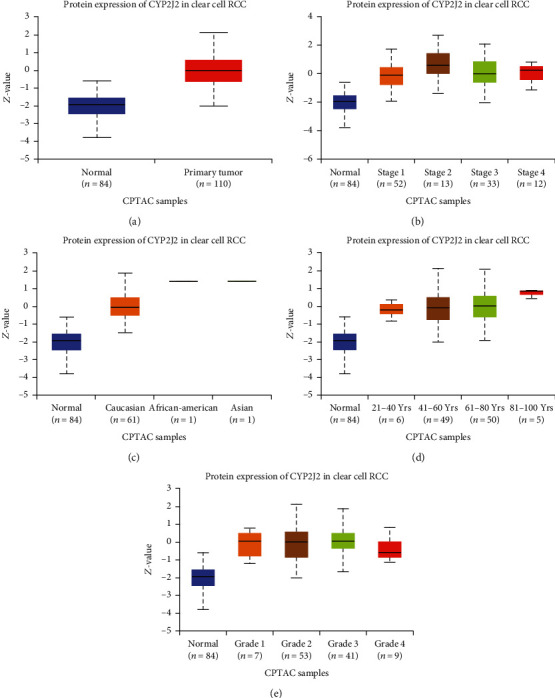
The protein expression level of CYP2J2 in ccRCC was evaluated using box plots in UALCAN database. (a–e) Box plots showing the association of sample types, cancer stage, patient's race, age, and tumor grade with CYP2J2 protein expression level in ccRCC. ccRCC: clear cell renal cell carcinoma; ccRCC=KIRC.

Expression levels of the CYP2J2 protein in KIRC were evaluated through the UALCAN database. Compared to matched normal tissues, expression levels of the CYP2J2 protein in KIRC were significantly elevated (Figure 7(a)). From the Oncomine, GEPIA, DriverDBV3, and UALCAN databases, expression levels of the CYP2J2 gene, transcript, and protein in KIRC were significantly elevated compared to matched normal tissues. Furthermore, to investigate the factors mediating the expression of the CYP2J2 protein, we evaluated the correlation between expression levels of the CYP2J2 protein and different clinical characteristics (Figures 7(b)–7(e)), findings of which were basically consistent with the expression trend of the CYP2J2 transcript. Given the transcript and protein levels of CYP2J2 were significantly elevated in the early stages of KIRC, it was concluded that CYP2J2 can be used as an early diagnostic marker for KIRC.

### 3.5. Promoter Methylation Level of CYP2J2 in KIRC

**Figure 8:**
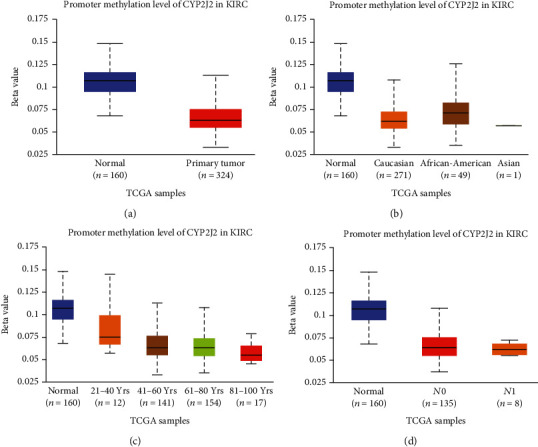
Promoter methylation levels of CYP2J2 in KIRC were evaluated by box plot using the UALCAN database. (a–d) Box plots showing the association of sample types, patient's race, age, and nodal metastasis status with promoter methylation level of CYP2J2 in KIRC.

We used the UALCAN database to evaluate promoter methylation level of CYP2J2 in KIRC. Compared to normal tissues, the methylation level of the CYP2J2 promoter in KIRC was lower (Figure 8(a)). Moreover, we evaluated the methylation level of the CYP2J2 promoter on the basis of different clinical characteristics, which showed a reverse trend with the expression level of the CYP2J2 transcript and protein (Figures 8(b)–8(d)). From the above results, we can infer that elevating the methylation level of the CYP2J2 promoter was likely to downregulate CYP2J2 expression in KIRC.

### 3.6. PPI Network of CYP2J2

**Figure 9:**
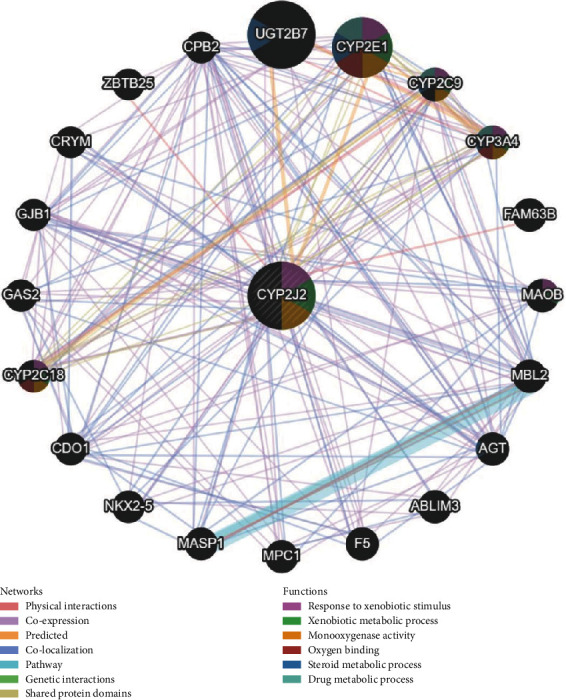
The PPI network of CYP2J2 was constructed by GeneMANIA. PPI: protein-protein interaction.

PPI showed functional networks between the CYP2J2 protein and other proteins. CYP2J2 was mainly enriched in responses to xenobiotic stimulus, xenobiotic metabolic process, monooxygenase activity, oxygen binding, steroid metabolic process, and drug metabolic process (Figure 9).

### 3.7. Functional Enrichment Analyses of CYP2J2

**Figure 10:**
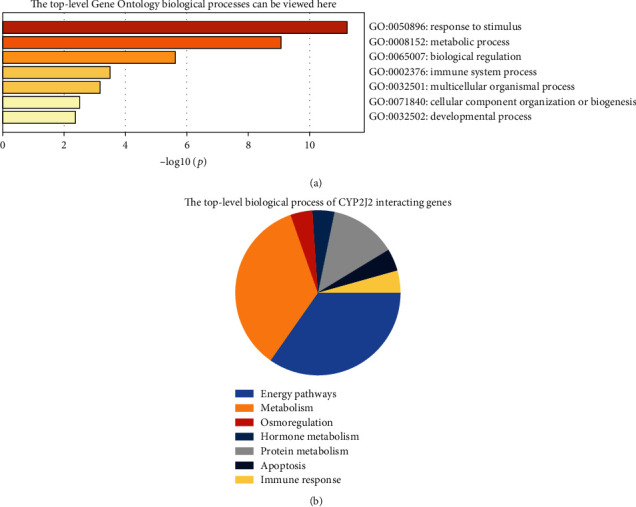
Biological processes involved in CYP2J2 interactive genes. (a) Heat map from Metascape showing the major biological processes involving the CYP2J2 interactive genes. (b) Pie chart from FunRich showing the major biological processes involving CYP2J2 interactive genes.

Biological processes of CYP2J2 interactive genes were evaluated by Metascape. We found that response to stimulus, metabolic process, biological regulation, immune system process, multicellular organismal process, cellular component organization or biogenesis, and developmental process was significantly regulated by these genes (Figure 10(a)), implying that CYP2J2 plays a very important role in metabolic process, immune regulation, response to stimulus, and cellular component organization or biogenesis. We also evaluated the biological processes of CYP2J2 interactive genes through FunRich (Figure 10(b)), and findings were very similar to those of Metascape. This further confirmed the biological processes involved in CYP2J2 interactive genes.

### 3.8. Correlation between CYP2J2 Expression Levels and Immune Cell Infiltration Levels in KIRC

**Figure 11:**
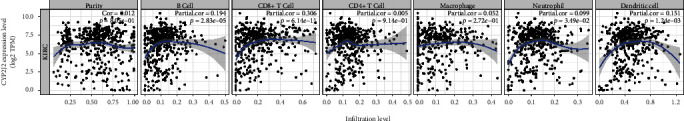
Relationships between CYP2J2 expression levels and immune cell infiltration levels in KIRC.

We used the TIMER web server to visualize the correlation between CYP2J2 gene expression levels and immune infiltration levels in KIRC. We found that the expression levels of CYP2J2 were positively correlated with B cells, CD8 + T cells, neutrophil, and dendritic cell infiltration levels in KIRC. However, it was not correlated with infiltration levels of CD4 + T cells and macrophages (Figure 11).

## 4. Discussion

Globally, KIRC is one of the most common malignancies, accounting for 80% of all kidney malignancies [24]. Due to the absence of a clear clinical biomarker for screening KIRC patients, approximately 15% of KIRC patients have metastatic tumors at the time of diagnosis [25]. Moreover, prognostic outcomes for patients with the same TNM stage and pathological grade may be different [26]. Therefore, it is imperative to identify biomarkers for the early diagnosis of KIRC and to determine their prognosis.

CYP2J2 is a member of the monooxygenase cytochrome P450 family, and it is highly expressed in the endothelium, myocardium, and kidneys [11]. It has been reported that CYP2J2 plays crucial roles in various diseases. For example, overexpression of CYP2J2 is beneficial in the treatment of diabetes [10] and in the reduction of cardiac hypertrophy [27]. However, CYP2J2 is significantly expressed in various tumors [28, 29], including KIRC, but it remains unknown if CYP2J2 overexpression in KIRC is beneficial or harmful.

Therefore, we investigated the correlation between CYP2J2 expression and KIRC using various databases, including Oncomine, GEPIA DriverDBv3, and TIMER databases. These databases showed that, among all tumors, CYP2J2 expression levels were significantly elevated in KIRC, implying that CYP2J2 may be a potential diagnostic marker for KIRC. Then, using UALCAN, GEPIA, OSkirc, and DriverDBv3 databases, we evaluated the effect of CYP2J2 expression on the survival time of KIRC patients. We found that overexpression of CYP2J2 prolonged the survival time of KIRC patients. These results suggest that CYP2J2 can be used as an important prognostic marker for KIRC patients. In addition, we reanalyzed the expression levels of the CYP2J2 gene and transcript in KIRC through UALCAN and found that their expression levels were significantly elevated in KIRC tumor tissues than in paracancerous normal samples. Moreover, expression levels of the CYP2J2 transcript in KIRC patients were higher in stage 1, grade 2, and *N*0 clinical features than in other clinical features. The expression trend of the CYP2J2 protein was basically consistent with that of the transcript, which implied that CYP2J2 can be used as an early diagnostic marker for KIRC patients. Some studies have reported that KIRC patients with lower grade, lower stage, and no lymph node metastasis are more likely to have longer survival outcomes than those with other clinical characteristics [5, 30], which was in agreement with our results. Taken together, these results indicate that CYP2J2 is a potential biomarker for the early diagnosis and prognosis of KIRC patients. Next, we evaluated the promoter methylation levels of CYP2J2 in KIRC through the UALCAN database, which exhibited a reverse trend with the expression levels of CYP2J2 transcript and protein. The result suggests that an enhanced promoter methylation level of CYP2J2 is likely to downregulate the CYP2J2 expression in KIRC patients.

To further determine the reasons why elevated expression levels of CYP2J2 are beneficial in the prognosis of KIRC patients, we evaluated the PPI network of CYP2J2 through GeneMANIA, while the biological processes involved in CYP2J2 interactive genes were evaluated through Metascape and FunRich. Since CYP2J2 was found to be involved in many biological processes, there were many possibilities for the specific reasons why CYP2J2 overexpression was beneficial in the prognosis of KIRC patients. We found that biological functions of CYP2J2 were correlated with immune processes; therefore, we postulated that this may be one of the reasons as to why KIRC patients with elevated CYP2J2 expression levels have better prognoses. Based on this assumption, we used TIMER to investigate the relationship between expression levels of CYP2J2 and immune cell infiltration levels in KIRC. The expression levels of CYP2J2 were positively correlated with infiltration levels of B cells, CD8 + T cells, neutrophils, and dendritic cells in KIRC. In addition, it has been reported that B cell infiltration in KIRC can prolong the cancer-specific survival [31], CD8 + T cell infiltration prolonged the OS outcomes of KIRC patients [32], and upregulation of the abundance of neutrophils was associated with a favorable prognosis of KIRC patients [33], while dendritic cells can effectively inhibit tumor recurrence and metastasis [34]. These results suggest that overexpression of CYP2J2 is beneficial to the prognosis of patients with KIRC by regulating the immune microenvironment of the tumor. However, CYP2J2-associated metabolic processes, apoptosis, and stimulus responses may also influence the prognosis of KIRC patients. Even though the mechanism by which CYP2J2 mediates the prognosis of KIRC patients has not been established, our findings suggest that CYP2J2 could be an important marker for the diagnosis and prognosis of KIRC patients and may be a potential therapeutic target for KIRC patients.

## 5. Conclusions

Expression levels of CYP2J2 in KIRC were most significant among various tumors and higher in stage 1, grade 2, and *N*0 clinical features than in other clinical features. Elevated expression of CYP2J2 can prolong survival outcomes in patients with KIRC. The biological processes involved in CYP2J2 interactive genes were significantly correlated with the responses of immune system processes. The expression levels of CYP2J2 were positively correlated with infiltration levels of B cells, CD8 + T cells, neutrophils, and dendritic cells in KIRC. CYP2J2 can be used as an early diagnostic marker and prognostic predictor of KIRC. Mechanistically, overexpression of CYP2J2 improves the prognosis of KIRC patients by regulating the immune microenvironment of the tumor. More studies are needed to confirm our findings and to facilitate the clinical use of CYP2J2 as a prognostic marker or as a therapeutic target for KIRC.

## Data Availability

The datasets analyzed for this study can be found in the Oncomine, GEPIA, DriverDBv3, OSkirc, UALCAN, GeneMANIA, Metascape, FunRich, and TIMER.
